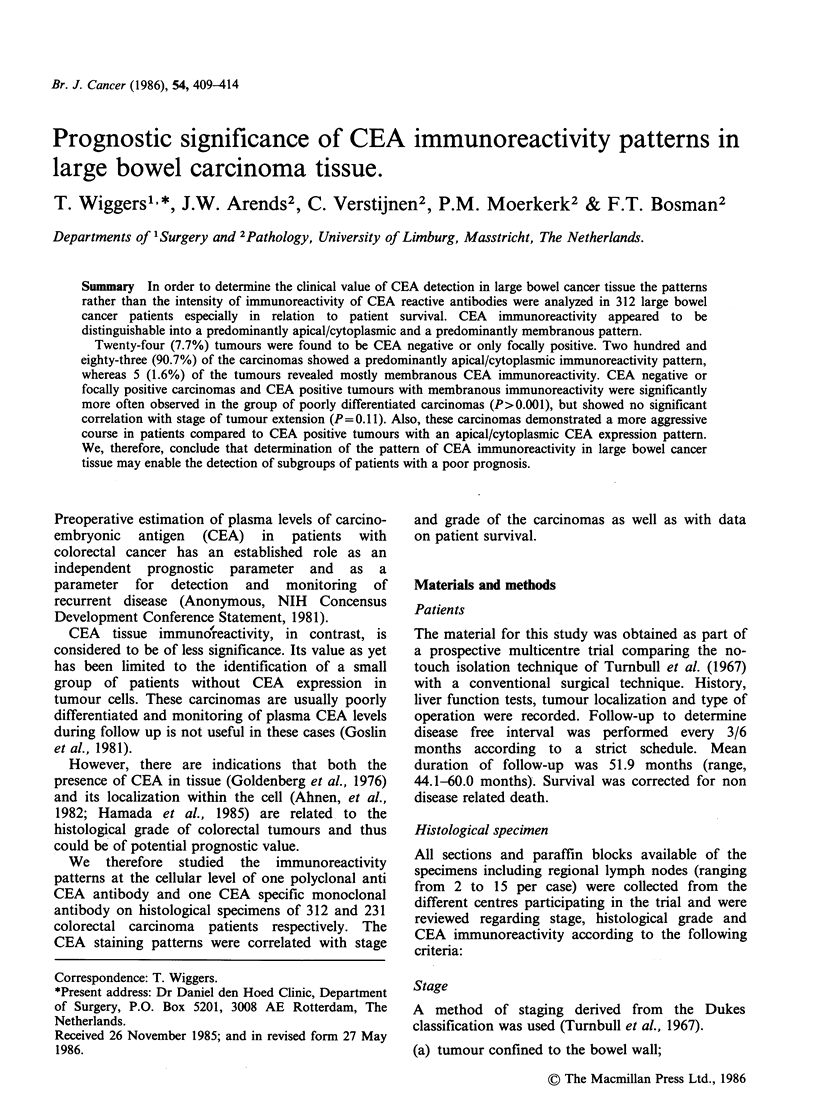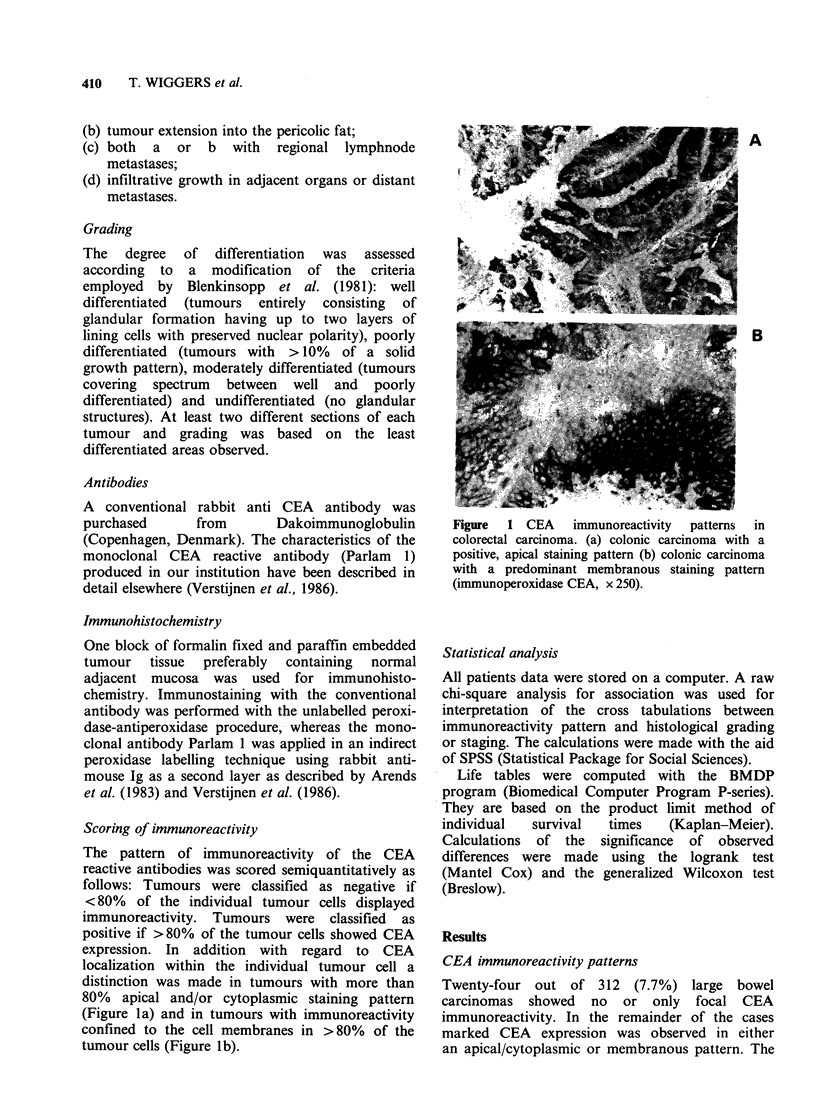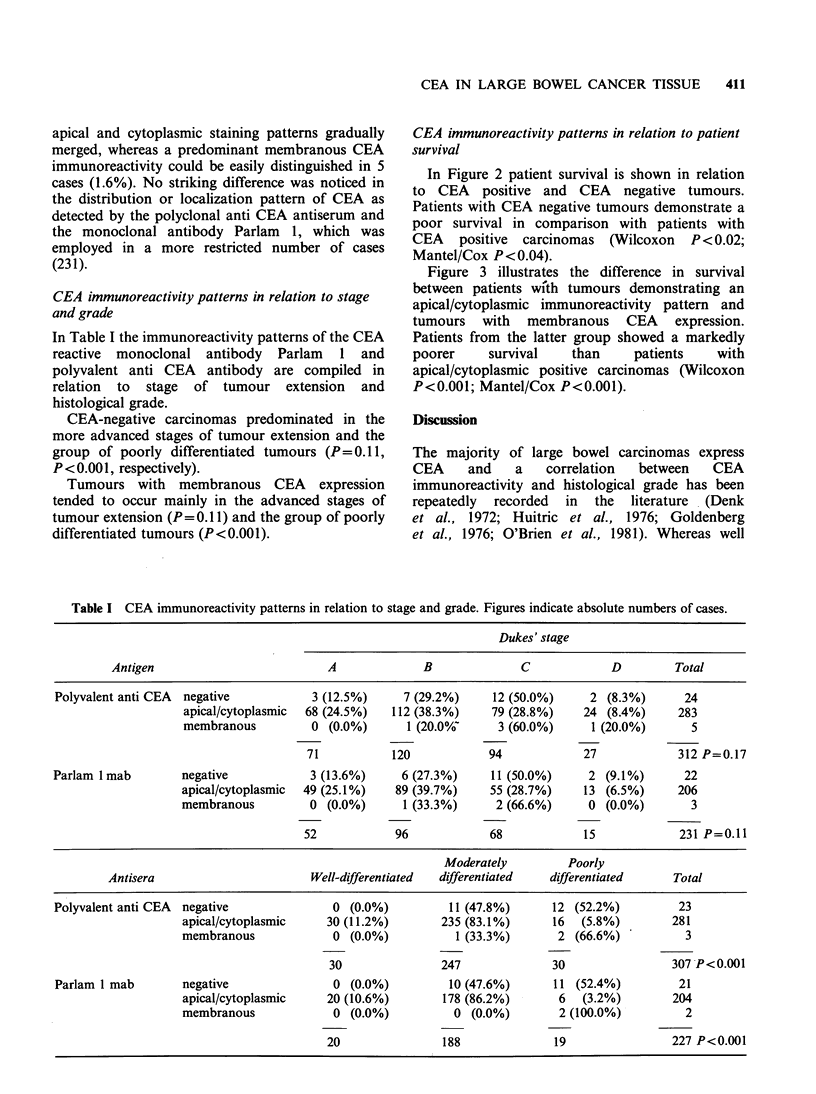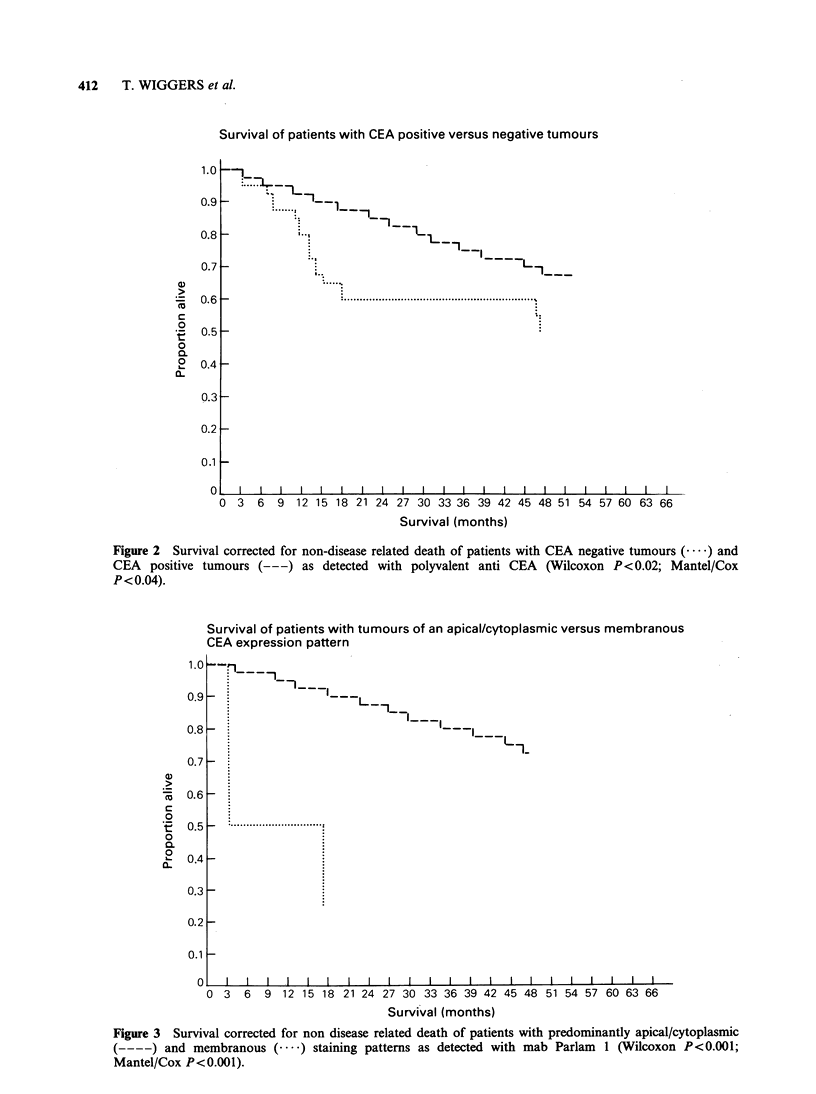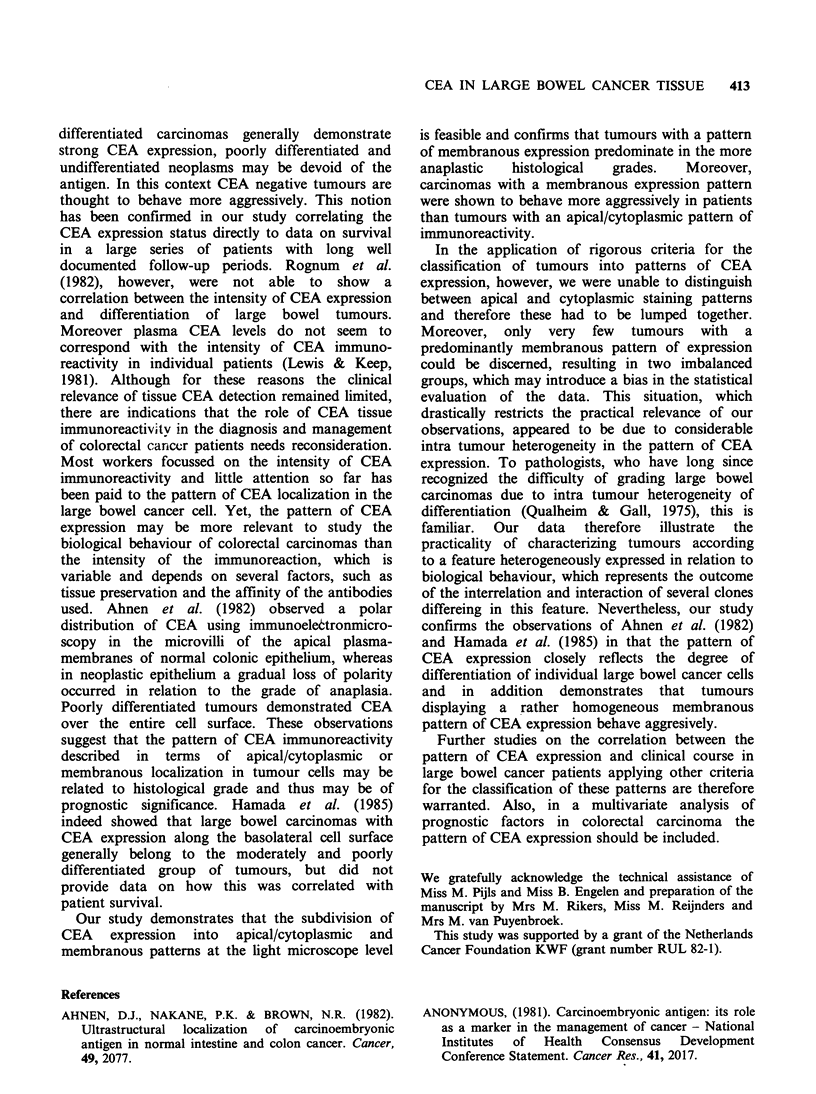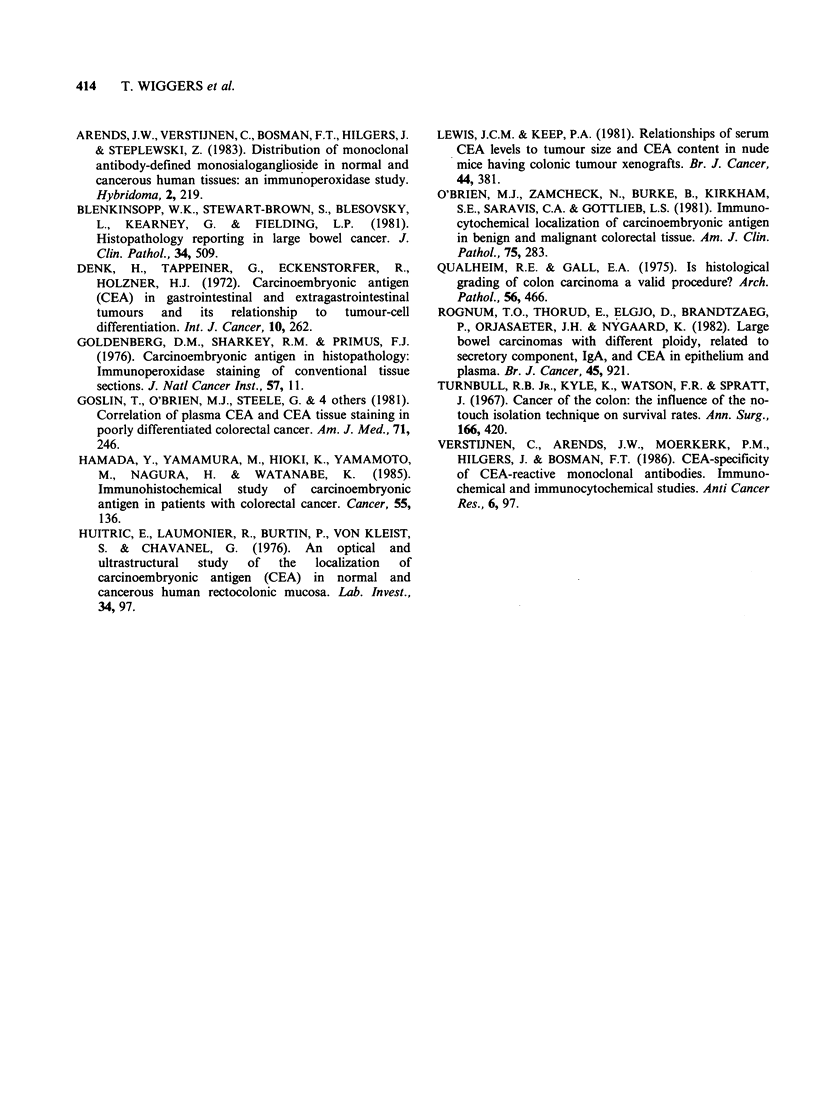# Prognostic significance of CEA immunoreactivity patterns in large bowel carcinoma tissue.

**DOI:** 10.1038/bjc.1986.191

**Published:** 1986-09

**Authors:** T. Wiggers, J. W. Arends, C. Verstijnen, P. M. Moerkerk, F. T. Bosman

## Abstract

**Images:**


*Br. J. Cancer* (1986), **54**, 409–414

### Prognostic significance of CEA immunoreactivity patterns in large bowel carcinoma tissue.

T. Wiggers[1,*], J.W. Arends[2], C. Verstijnen[2], P.M. Moerkerk[2] & F.T. Bosman[2]

*Departments of [1]Surgery and [2]Pathology, University of Limburg, Masstricht, The Netherlands.*

**Summary** In order to determine the clinical value of CEA detection in large bowel cancer tissue the patterns rather than the intensity of immunoreactivity of CEA reactive antibodies were analyzed in 312 large bowel cancer patients especially in relation to patient survival. CEA immunoreactivity appeared to be distinguishable into a predominantly apical/cytoplasmic and a predominantly membranous pattern.

Twenty-four (7.7%) tumours were found to be CEA negative or only focally positive. Two hundred and eighty-three (90.7%) of the carcinomas showed a predominantly apical/cytoplasmic immunoreactivity pattern, whereas 5 (1.6%) of the tumours revealed mostly membranous CEA immunoreactivity. CEA negative or focally positive carcinomas and CEA positive tumours with membranous immunoreactivity were significantly more often observed in the group of poorly differentiated carcinomas ($P>0.001$), but showed no significant correlation with stage of tumour extension ($P=0.11$). Also, these carcinomas demonstrated a more aggressive course in patients compared to CEA positive tumours with an apical/cytoplasmic CEA expression pattern. We, therefore, conclude that determination of the pattern of CEA immunoreactivity in large bowel cancer tissue may enable the detection of subgroups of patients with a poor prognosis.

Preoperative estimation of plasma levels of carcinoembryonic antigen (CEA) in patients with colorectal cancer has an established role as an independent prognostic parameter and as a parameter for detection and monitoring of recurrent disease (Anonymous, NIH Concensus Development Conference Statement, 1981).

CEA tissue immunoreactivity, in contrast, is considered to be of less significance. Its value as yet has been limited to the identification of a small group of patients without CEA expression in tumour cells. These carcinomas are usually poorly differentiated and monitoring of plasma CEA levels during follow up is not useful in these cases (Goslin *et al.*, 1981).

However, there are indications that both the presence of CEA in tissue (Goldenberg *et al.*, 1976) and its localization within the cell (Ahnen, *et al.*, 1982; Hamada *et al.*, 1985) are related to the histological grade of colorectal tumours and thus could be of potential prognostic value.

We therefore studied the immunoreactivity patterns at the cellular level of one polyclonal anti CEA antibody and one CEA specific monoclonal antibody on histological specimens of 312 and 231 colorectal carcinoma patients respectively. The CEA staining patterns were correlated with stage and grade of the carcinomas as well as with data on patient survival.

Correspondence: T. Wiggers.
*Present address: Dr Daniel den Hoed Clinic, Department of Surgery, P.O. Box 5201, 3008 AE Rotterdam, The Netherlands.
Received 26 November 1985; and in revised form 27 May 1986.

### Materials and methods

### *Patients*

The material for this study was obtained as part of a prospective multicentre trial comparing the no-touch isolation technique of Turnbull *et al.* (1967) with a conventional surgical technique. History, liver function tests, tumour localization and type of operation were recorded. Follow-up to determine disease free interval was performed every 3/6 months according to a strict schedule. Mean duration of follow-up was 51.9 months (range, 44.1–60.0 months). Survival was corrected for non disease related death.

### *Histological specimen*

All sections and paraffin blocks available of the specimens including regional lymph nodes (ranging from 2 to 15 per case) were collected from the different centres participating in the trial and were reviewed regarding stage, histological grade and CEA immunoreactivity according to the following criteria:

### *Stage*

A method of staging derived from the Dukes classification was used (Turnbull *et al.*, 1967).

(a) tumour confined to the bowel wall;

© The Macmillan Press Ltd., 1986

(b) tumour extension into the pericolic fat;
(c) both a or b with regional lymphnode metastases;
(d) infiltrative growth in adjacent organs or distant metastases.

*Grading*

The degree of differentiation was assessed according to a modification of the criteria employed by Blenkinsopp *et al.* (1981): well differentiated (tumours entirely consisting of glandular formation having up to two layers of lining cells with preserved nuclear polarity), poorly differentiated (tumours with >10% of a solid growth pattern), moderately differentiated (tumours covering spectrum between well and poorly differentiated) and undifferentiated (no glandular structures). At least two different sections of each tumour and grading was based on the least differentiated areas observed.

*Antibodies*

A conventional rabbit anti CEA antibody was purchased from Dakoimmunoglobulin (Copenhagen, Denmark). The characteristics of the monoclonal CEA reactive antibody (Parlam 1) produced in our institution have been described in detail elsewhere (Verstijnen *et al.*, 1986).

*Immunohistochemistry*

One block of formalin fixed and paraffin embedded tumour tissue preferably containing normal adjacent mucosa was used for immunohistochemistry. Immunostaining with the conventional antibody was performed with the unlabelled peroxidase-antiperoxidase procedure, whereas the monoclonal antibody Parlam 1 was applied in an indirect peroxidase labelling technique using rabbit anti-mouse Ig as a second layer as described by Arends *et al.* (1983) and Verstijnen *et al.* (1986).

*Scoring of immunoreactivity*

The pattern of immunoreactivity of the CEA reactive antibodies was scored semiquantitatively as follows: Tumours were classified as negative if <80% of the individual tumour cells displayed immunoreactivity. Tumours were classified as positive if >80% of the tumour cells showed CEA expression. In addition with regard to CEA localization within the individual tumour cell a distinction was made in tumours with more than 80% apical and/or cytoplasmic staining pattern (Figure 1a) and in tumours with immunoreactivity confined to the cell membranes in >80% of the tumour cells (Figure 1b).

**Figure 1** CEA immunoreactivity patterns in colorectal carcinoma. (a) colonic carcinoma with a positive, apical staining pattern (b) colonic carcinoma with a predominant membranous staining pattern (immunoperoxidase CEA, ×250).

*Statistical analysis*

All patients data were stored on a computer. A raw chi-square analysis for association was used for interpretation of the cross tabulations between immunoreactivity pattern and histological grading or staging. The calculations were made with the aid of SPSS (Statistical Package for Social Sciences).

Life tables were computed with the BMDP program (Biomedical Computer Program P-series). They are based on the product limit method of individual survival times (Kaplan–Meier). Calculations of the significance of observed differences were made using the logrank test (Mantel Cox) and the generalized Wilcoxon test (Breslow).

### Results

*CEA immunoreactivity patterns*

Twenty-four out of 312 (7.7%) large bowel carcinomas showed no or only focal CEA immunoreactivity. In the remainder of the cases marked CEA expression was observed in either an apical/cytoplasmic or membranous pattern. The apical and cytoplasmic staining patterns gradually merged, whereas a predominant membranous CEA immunoreactivity could be easily distinguished in 5 cases (1.6%). No striking difference was noticed in the distribution or localization pattern of CEA as detected by the polyclonal anti CEA antiserum and the monoclonal antibody Parlam 1, which was employed in a more restricted number of cases (231).

*CEA immunoreactivity patterns in relation to stage and grade*

In Table I the immunoreactivity patterns of the CEA reactive monoclonal antibody Parlam 1 and polyvalent anti CEA antibody are compiled in relation to stage of tumour extension and histological grade.

CEA-negative carcinomas predominated in the more advanced stages of tumour extension and the group of poorly differentiated tumours ($P=0.11$, $P<0.001$, respectively).

Tumours with membranous CEA expression tended to occur mainly in the advanced stages of tumour extension ($P=0.11$) and the group of poorly differentiated tumours ($P<0.001$).

*CEA immunoreactivity patterns in relation to patient survival*

In Figure 2 patient survival is shown in relation to CEA positive and CEA negative tumours. Patients with CEA negative tumours demonstrate a poor survival in comparison with patients with CEA positive carcinomas (Wilcoxon $P<0.02$; Mantel/Cox $P<0.04$).

Figure 3 illustrates the difference in survival between patients with tumours demonstrating an apical/cytoplasmic immunoreactivity pattern and tumours with membranous CEA expression. Patients from the latter group showed a markedly poorer survival than patients with apical/cytoplasmic positive carcinomas (Wilcoxon $P<0.001$; Mantel/Cox $P<0.001$).

### Discussion

The majority of large bowel carcinomas express CEA and a correlation between CEA immunoreactivity and histological grade has been repeatedly recorded in the literature (Denk *et al.*, 1972; Huitric *et al.*, 1976; Goldenberg *et al.*, 1976; O'Brien *et al.*, 1981). Whereas well

**Table I** CEA immunoreactivity patterns in relation to stage and grade. Figures indicate absolute numbers of cases.

| | | *Dukes' stage* | | | | |
|---|---|---|---|---|---|---|
| *Antigen* | | *A* | *B* | *C* | *D* | *Total* |
| Polyvalent anti CEA | negative | 3 (12.5%) | 7 (29.2%) | 12 (50.0%) | 2 (8.3%) | 24 |
| | apical/cytoplasmic | 68 (24.5%) | 112 (38.3%) | 79 (28.8%) | 24 (8.4%) | 283 |
| | membranous | 0 (0.0%) | 1 (20.0% | 3 (60.0%) | 1 (20.0%) | 5 |
| | | 71 | 120 | 94 | 27 | 312 $P=0.17$ |
| Parlam 1 mab | negative | 3 (13.6%) | 6 (27.3%) | 11 (50.0%) | 2 (9.1%) | 22 |
| | apical/cytoplasmic | 49 (25.1%) | 89 (39.7%) | 55 (28.7%) | 13 (6.5%) | 206 |
| | membranous | 0 (0.0%) | 1 (33.3%) | 2 (66.6%) | 0 (0.0%) | 3 |
| | | 52 | 96 | 68 | 15 | 231 $P=0.11$ |

| *Antisera* | | *Well-differentiated* | *Moderately differentiated* | *Poorly differentiated* | *Total* |
|---|---|---|---|---|---|
| Polyvalent anti CEA | negative | 0 (0.0%) | 11 (47.8%) | 12 (52.2%) | 23 |
| | apical/cytoplasmic | 30 (11.2%) | 235 (83.1%) | 16 (5.8%) | 281 |
| | membranous | 0 (0.0%) | 1 (33.3%) | 2 (66.6%) | 3 |
| | | 30 | 247 | 30 | 307 $P<0.001$ |
| Parlam 1 mab | negative | 0 (0.0%) | 10 (47.6%) | 11 (52.4%) | 21 |
| | apical/cytoplasmic | 20 (10.6%) | 178 (86.2%) | 6 (3.2%) | 204 |
| | membranous | 0 (0.0%) | 0 (0.0%) | 2 (100.0%) | 2 |
| | | 20 | 188 | 19 | 227 $P<0.001$ |

**Figure 2** Survival corrected for non-disease related death of patients with CEA negative tumours (····) and CEA positive tumours (–––) as detected with polyvalent anti CEA (Wilcoxon $P<0.02$; Mantel/Cox $P<0.04$).

**Figure 3** Survival corrected for non disease related death of patients with predominantly apical/cytoplasmic (––––) and membranous (····) staining patterns as detected with mab Parlam 1 (Wilcoxon $P<0.001$; Mantel/Cox $P<0.001$).

differentiated carcinomas generally demonstrate strong CEA expression, poorly differentiated and undifferentiated neoplasms may be devoid of the antigen. In this context CEA negative tumours are thought to behave more aggressively. This notion has been confirmed in our study correlating the CEA expression status directly to data on survival in a large series of patients with long well documented follow-up periods. Rognum *et al.* (1982), however, were not able to show a correlation between the intensity of CEA expression and differentiation of large bowel tumours. Moreover plasma CEA levels do not seem to correspond with the intensity of CEA immunoreactivity in individual patients (Lewis & Keep, 1981). Although for these reasons the clinical relevance of tissue CEA detection remained limited, there are indications that the role of CEA tissue immunoreactivity in the diagnosis and management of colorectal cancer patients needs reconsideration. Most workers focussed on the intensity of CEA immunoreactivity and little attention so far has been paid to the pattern of CEA localization in the large bowel cancer cell. Yet, the pattern of CEA expression may be more relevant to study the biological behaviour of colorectal carcinomas than the intensity of the immunoreaction, which is variable and depends on several factors, such as tissue preservation and the affinity of the antibodies used. Ahnen *et al.* (1982) observed a polar distribution of CEA using immunoelectronmicroscopy in the microvilli of the apical plasmamembranes of normal colonic epithelium, whereas in neoplastic epithelium a gradual loss of polarity occurred in relation to the grade of anaplasia. Poorly differentiated tumours demonstrated CEA over the entire cell surface. These observations suggest that the pattern of CEA immunoreactivity described in terms of apical/cytoplasmic or membranous localization in tumour cells may be related to histological grade and thus may be of prognostic significance. Hamada *et al.* (1985) indeed showed that large bowel carcinomas with CEA expression along the basolateral cell surface generally belong to the moderately and poorly differentiated group of tumours, but did not provide data on how this was correlated with patient survival.

Our study demonstrates that the subdivision of CEA expression into apical/cytoplasmic and membranous patterns at the light microscope level is feasible and confirms that tumours with a pattern of membranous expression predominate in the more anaplastic histological grades. Moreover, carcinomas with a membranous expression pattern were shown to behave more aggressively in patients than tumours with an apical/cytoplasmic pattern of immunoreactivity.

In the application of rigorous criteria for the classification of tumours into patterns of CEA expression, however, we were unable to distinguish between apical and cytoplasmic staining patterns and therefore these had to be lumped together. Moreover, only very few tumours with a predominantly membranous pattern of expression could be discerned, resulting in two imbalanced groups, which may introduce a bias in the statistical evaluation of the data. This situation, which drastically restricts the practical relevance of our observations, appeared to be due to considerable intra tumour heterogeneity in the pattern of CEA expression. To pathologists, who have long since recognized the difficulty of grading large bowel carcinomas due to intra tumour heterogeneity of differentiation (Qualheim & Gall, 1975), this is familiar. Our data therefore illustrate the practicality of characterizing tumours according to a feature heterogeneously expressed in relation to biological behaviour, which represents the outcome of the interrelation and interaction of several clones differeing in this feature. Nevertheless, our study confirms the observations of Ahnen *et al.* (1982) and Hamada *et al.* (1985) in that the pattern of CEA expression closely reflects the degree of differentiation of individual large bowel cancer cells and in addition demonstrates that tumours displaying a rather homogeneous membranous pattern of CEA expression behave aggresively.

Further studies on the correlation between the pattern of CEA expression and clinical course in large bowel cancer patients applying other criteria for the classification of these patterns are therefore warranted. Also, in a multivariate analysis of prognostic factors in colorectal carcinoma the pattern of CEA expression should be included.

We gratefully acknowledge the technical assistance of Miss M. Pijls and Miss B. Engelen and preparation of the manuscript by Mrs M. Rikers, Miss M. Reijnders and Mrs M. van Puyenbroek.

This study was supported by a grant of the Netherlands Cancer Foundation KWF (grant number RUL 82-1).

### References

AHNEN, D.J., NAKANE, P.K. & BROWN, N.R. (1982). Ultrastructural localization of carcinoembryonic antigen in normal intestine and colon cancer. *Cancer*, **49**, 2077.

ANONYMOUS, (1981). Carcinoembryonic antigen: its role as a marker in the management of cancer – National Institutes of Health Consensus Development Conference Statement. *Cancer Res.*, **41**, 2017.

ARENDS, J.W., VERSTIJNEN, C., BOSMAN, F.T., HILGERS, J. & STEPLEWSKI, Z. (1983). Distribution of monoclonal antibody-defined monosialoganglioside in normal and cancerous human tissues: an immunoperoxidase study. *Hybridoma*, **2**, 219.

BLENKINSOPP, W.K., STEWART-BROWN, S., BLESOVSKY, L., KEARNEY, G. & FIELDING, L.P. (1981). Histopathology reporting in large bowel cancer. *J. Clin. Pathol.*, **34**, 509.

DENK, H., TAPPEINER, G., ECKENSTORFER, R., HOLZNER, H.J. (1972). Carcinoembryonic antigen (CEA) in gastrointestinal and extragastrointestinal tumours and its relationship to tumour-cell differentiation. *Int. J. Cancer*, **10**, 262.

GOLDENBERG, D.M., SHARKEY, R.M. & PRIMUS, F.J. (1976). Carcinoembryonic antigen in histopathology: Immunoperoxidase staining of conventional tissue sections. *J. Natl Cancer Inst.*, **57**, 11.

GOSLIN, T., O'BRIEN, M.J., STEELE, G. & 4 others (1981). Correlation of plasma CEA and CEA tissue staining in poorly differentiated colorectal cancer. *Am. J. Med.*, **71**, 246.

HAMADA, Y., YAMAMURA, M., HIOKI, K., YAMAMOTO, M., NAGURA, H. & WATANABE, K. (1985). Immunohistochemical study of carcinoembryonic antigen in patients with colorectal cancer. *Cancer*, **55**, 136.

HUITRIC, E., LAUMONIER, R., BURTIN, P., VON KLEIST, S. & CHAVANEL, G. (1976). An optical and ultrastructural study of the localization of carcinoembryonic antigen (CEA) in normal and cancerous human rectocolonic mucosa. *Lab. Invest.*, **34**, 97.

LEWIS, J.C.M. & KEEP, P.A. (1981). Relationships of serum CEA levels to tumour size and CEA content in nude mice having colonic tumour xenografts. *Br. J. Cancer*, **44**, 381.

O'BRIEN, M.J., ZAMCHECK, N., BURKE, B., KIRKHAM, S.E., SARAVIS, C.A. & GOTTLIEB, L.S. (1981). Immunocytochemical localization of carcinoembryonic antigen in benign and malignant colorectal tissue. *Am. J. Clin. Pathol.*, **75**, 283.

QUALHEIM, R.E. & GALL, E.A. (1975). Is histological grading of colon carcinoma a valid procedure? *Arch. Pathol.*, **56**, 466.

ROGNUM, T.O., THORUD, E., ELGJO, D., BRANDTZAEG, P., ORJASAETER, J.H. & NYGAARD, K. (1982). Large bowel carcinomas with different ploidy, related to secretory component, IgA, and CEA in epithelium and plasma. *Br. J. Cancer*, **45**, 921.

TURNBULL, R.B. JR., KYLE, K., WATSON, F.R. & SPRATT, J. (1967). Cancer of the colon: the influence of the no-touch isolation technique on survival rates. *Ann. Surg.*, **166**, 420.

VERSTIJNEN, C., ARENDS, J.W., MOERKERK, P.M., HILGERS, J. & BOSMAN, F.T. (1986). CEA-specificity of CEA-reactive monoclonal antibodies. Immunochemical and immunocytochemical studies. *Anti Cancer Res.*, **6**, 97.